# Open access is good for you!

**DOI:** 10.1107/S1600536814014457

**Published:** 2014-07-19

**Authors:** Luc Van Meervelt

**Affiliations:** a Chemistry Department, KU Leuven, Celestijnenlaan 200F, B-3001 Leuven (Heverlee), Belgium

**Keywords:** crystallography, editorial, Acta E

The introduction of open access has greatly influenced the way researchers publish their results and consult those of their colleagues. Basically two routes have been designed for open access. By using open-access repositories, the green route makes published articles and results freely available on the internet, typically after permission and an embargo period decided by the journal publisher. In most cases this archiving is done by institutions and universities. In the second so-called gold route, authors publish in an open-access journal. Two types exist here, a fully open-access journal or a hydrid journal containing conventional and open-access articles, but for both types a publication fee is charged in most cases. The Directory of Open Access Journals (DOAJ), an online directory that indexes and provides access to open-access journals, currently contains about 9900 journal titles. 

The IUCr Executive Committee decided during its meeting in Leuven in August 2006 to go for the true open-access model in the case of *Acta Crystallographica Section E*. This choice has proven to be very successful, resulting in the annual publication of about 4000 fully open-access papers. The recently announced transformation of *Acta Crystallographica Section E: Structure Reports Online* to *Crystallographic Communications* and the introduction of two new article types (longer *Research Communications* and short-format *Data Reports*) will not however change the open-access policy. 

For researchers the most important advantages of open access are the increased visibility and accessibility of their data and results. Usage and access to research literature becomes free and much easier, which is certainly further enhanced by the worldwide development of the internet. In times when research institutes and universities – and not only in lower-income countries – can allocate less resources for subscriptions to international journals, this is an important benefit for all researchers. As a consequence, open-access articles are more widely read, cited sooner and achieve higher citation rates. 

The shift of traditional publishing to open access also forces publishers to adopt new business models. The income from traditional subscriptions to journals or pay-per-view services for individual articles is gradually being replaced by payment of a processing fee by the authors or their institutions and funding agencies. Especially during the transition period, this causes the impression that one is paying double. Policy makers can play an important role here to shorten this transition period and to resolve the ambiguity. 

Nowadays many universities argue the benefits of open access for their researchers and encourage them to publish in open-access journals, which may help to fulfil their mission of creating and disseminating knowledge. The League of European Research Universities (LERU) has a specific roadmap towards open access to assist LERU members and other European universities who wish to facilitate open access. Research funding agencies also recommend open-access publishing as a way to ensure the maximal impact of their support. Open access is mandatory for all research publications that result from projects funded by the European Commission Horizon 2020 programme; this has a seven-year budget of nearly EUR 80 billion and also includes reimbursement of author processing charges. 

The Editorial Board of *Acta Cryst. E* looks forward to receiving submissions in the new *Research Communications* and *Data Reports* formats from the crystallographic community. By offering gold-route open access for a very modest open-access fee, the IUCr hopes to serve the crystallographic community and related fields. By disseminating your high-quality data and reporting your scientific findings, we hope to further increase the visibility and impact of our crystallographic research. Open access is good for all of us!

